# A Single-center Experience Comparing First- Versus Second-generation Insertable Cardiac Monitors in Pediatric Patients

**DOI:** 10.19102/icrm.2022.130605

**Published:** 2022-06-15

**Authors:** Nathan Miller, Lisa Roelle, Dean Lorimer, Aarti S. Dalal, William B. Orr, George F. Van Hare, Jennifer N. Avari Silva

**Affiliations:** ^1^Electrophysiology Laboratory, St. Louis Children’s Hospital, St. Louis, MO, USA; ^2^Division of Pediatric Cardiology, Department of Pediatrics, Washington University School of Medicine, St. Louis, MO, USA; ^3^Department of Biomedical Engineering, Washington University McKelvey School of Engineering, St. Louis, MO, USA

**Keywords:** Arrhythmias, inherited arrhythmia syndromes, insertable cardiac monitor, palpitations, pediatric electrophysiology, syncope

## Abstract

Insertable cardiac monitors (ICMs) have undergone advancements in size and functionality over the past decade, resulting in the introduction of small, easily insertable devices capable of long-term remote monitoring. We define first-generation ICMs as implantable cardiac monitoring devices that require an incision and surgical creation of a subcutaneous pocket and second-generation ICMs as devices implanted using a custom-made tool for subcutaneous insertion, respectively. The aim of this study was to understand the differences between first- and second-generation pediatric ICM implants, implant indications, and time to diagnosis. We performed a retrospective, single-center chart review of patients who underwent ICM implantation from 2009–2019, spanning a 5-year course of first-generation ICM implantations and 5-year course of second-generation ICM implantations. Demographic data, past medical history, implant indication, and time to diagnosis were obtained. A total of 208 patients were identified over the 10-year time period, including 38 (18%) who underwent implantation with a first-generation device and 170 (82%) who underwent implantation with a second-generation device. Implant indications for first-generation ICMs included syncope (71%), palpitations (16%), inherited arrhythmia syndrome (IAS) management (5%), and premature ventricular contractions/ventricular tachycardia (VT) (8%); implant indications for second-generation ICMs included syncope (48%), palpitations (19%), IAS management (40%), premature ventricular contractions/VT (11%), atrial fibrillation (2%), tachycardia (3%), and heart block (0.5%). The average time to diagnosis was 38 weeks for patients with first-generation devices and 55 weeks for those with second-generation devices. With innovations in ICM technologies, there are expanding indications for ICM implantation in pediatric patients for long-term monitoring, specifically regarding the management of IAS patients.

## Introduction

Advances in cardiac monitoring for patients with paroxysmal symptoms have evolved from fully external devices to wearable devices, including on-body patch devices and watches. A careful assessment of the patient’s symptoms, including the character, frequency, and duration, can help determine the optimal device for establishing the symptom–rhythm correlation.^1–4^ Various external monitoring devices are useful in quickly yielding a diagnosis in patients with frequent or long-duration symptoms. However, readily available external monitors and wearables may not be ideally suited for pediatric patients who are unable to articulate symptoms, have infrequent symptoms, or require longer-term cardiac monitoring.^5^

Insertable cardiac monitors (ICMs) are widely used in adult and pediatric patients who require chronic arrhythmia burden assessment or symptom–rhythm correlation with infrequent symptoms.^6,7^ Technical advances in devices have resulted in smaller, easily implantable or insertable devices with longer battery lives that can provide remote wireless monitoring over a multiyear timespan.^8^ ICMs have proven to be useful in pediatric patients^9–12^ and in patients with inherited arrhythmia syndromes (IASs), allowing for the documentation of rhythm during symptom events, arrhythmia surveillance, return to sports/reassurance, and documentation of subclinical arrhythmias.^5,13^ Current common uses for ICM implantation in pediatric patients include diagnosis of arrhythmias in patients with syncope or palpitations, assessment of premature ventricular complex (PVC) or ventricular tachycardia (VT) burden, and surveillance in patients with IASs.^8,14,15^

The aim of this single-center study was to describe the frequency and clinical indications for implantation of first-generation versus second-generation ICMs within a pediatric population. The diagnostic yield of the ICMs was measured by assessing the time to diagnosis and time to intervention. There was an increase in the total number of devices implanted per year and in the number of second-generation ICMs implanted per year compared to first-generation devices over the duration.

## Methods

After obtaining approval from the Washington University School of Medicine Institutional Review Board, a retrospective, single-center chart review was performed spanning the years 2009–2019. At our institution, the transition from first- to second-generation ICMs occurred in 2014. First-generation ICMs were defined as implantable cardiac monitoring devices that require an incision and surgical creation of a subcutaneous pocket. Second-generation ICMs were defined as insertable devices that have a smaller form factor defined as hardware design features that guide and define the size, shape, and other physical specifications of electronic components. Newer second-generation ICMs use a custom-made tool for subcutaneous insertion.

During the chart review, the following information was collected from the medical records: patient demographics (including name and age), date of ICM implantation, make and model of the device, clinical indication for ICM placement, patient diagnosis, current status of ICM (including if the patient has been lost to follow-up or the ICM is still in place), time to diagnosis, occurrence of patient intervention based on ICM data as well as time to intervention, and any complications. The time to diagnosis was defined as the time from the date of implantation to the date of transmission received that yielded a diagnosis. Interventions included pacemaker implantation, implantable cardioverter-defibrillator (ICD) placement, electrophysiology study (EPS) with or without catheter ablation, or replacement of ICM due to battery depletion. The time to intervention was defined as the time from the date of ICM implantation to the date of diagnostic/therapeutic procedure.

Patients with IAS as their primary diagnosis, regardless of symptoms, included those diagnosed with long QT syndrome (LQTS), catecholaminergic polymorphic VT, Brugada syndrome, and hypertrophic cardiomyopathy. All IAS patients were phenotype-positive, regardless of the genotype status (which may include a genotype-positive, genotype-negative, or genotype-unknown status).

### Statistical analysis

Descriptive data are presented as mean ± standard deviation values or percentages as appropriate. Two-proportion Z tests were performed to assess the proportion differences between populations. *P* < .05 was used for statistical significance.

## Results

### Demographics

**Figure 1:**
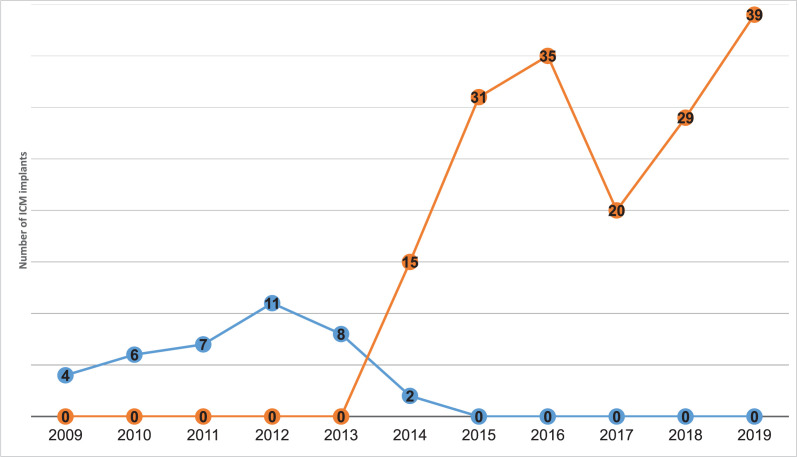
First- versus second-generation implantable cardiac monitors (ICMs) implanted by year. This graph represents the absolute number of ICM implants by year, with the blue line demonstrating first-generation ICMs and the orange line demonstrating second-generation ICMs. During the transition year of 2014, when the study institution moved from first- to second-generation ICMs, there were more second-generation ICMs than first-generation ICMs implanted. Also notable is that yearly implants were notably higher with second-generation ICMs.

A total of 208 patients were identified during the study period; of these, 38 patients had first-generation ICMs and 170 patients had second-generation ICMs **(Table 1)**. There was no significant difference in sex, age, weight, or height of the patients implanted with first-generation versus second-generation ICMs. There was an increase in the total number of devices implanted per year, as well as in the number of second-generation ICMs implanted per year compared to first-generation devices, over the duration of the study **(Figure 1)**.

### Indication for implant

For first-generation ICM implants, the most common indication for implantation was syncope/near-syncope (n = 27, 71%) followed by palpitations (n = 6, 16%), PVCs/VT (n = 3, 8%), and IASs (n = 2, 5%). In contrast, second-generation ICMs were most commonly implanted for syncope/near syncope (n = 81, 48%), followed by IASs (n = 40, 24%); palpitations (n = 32, 19%); PVCs/VT (n = 11, 6%); tachycardia, which was defined as symptomatic tachycardia with the inability to diagnose arrhythmia from an external cardiac monitor (n = 3, 2%); atrial fibrillation (n = 2, 1%); and heart block (n = 1, 0.5%) **(Table 2)**. There was a significant decrease in the use of second-generation ICMs for syncope/near-syncope (71% first-generation vs. 48% second-generation, *P* = .009) and a significant increase in the use of second-generation ICMs in patients with IASs (5% first-generation vs. 25% second-generation, *P* = .01).

### Follow-up

**Figure 2:**
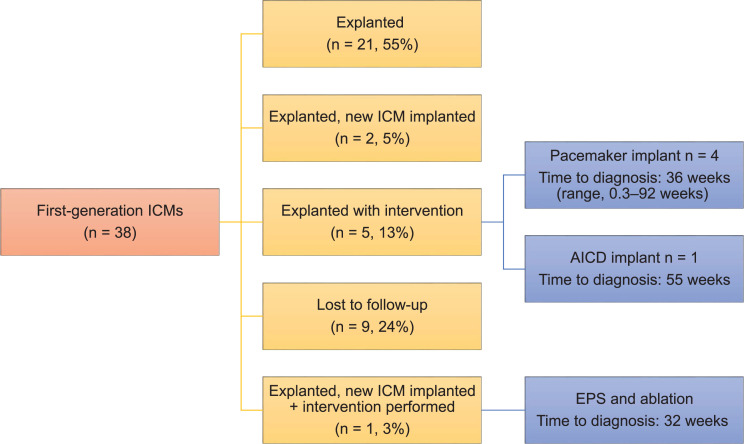
Follow-up of first-generation implantable cardiac monitors (ICMs). A total of 38 first-generation ICMs were implanted between 2009–2014. Of these devices, 55% were explanted, 24% were lost to follow-up, 5% were explanted with a new ICM implanted, and 16% were explanted with an intervention or a new ICM implant with an intervention. The interventions performed included a pacemaker implant, an automatic implantable cardioverter-defibrillator implant, and an electrophysiology study + ablation. *Abbreviations*: AICD, automatic implantable cardioverter-defibrillator; EPS, electrophysiology study; ICM, insertable cardiac monitor.

**Figure 3:**
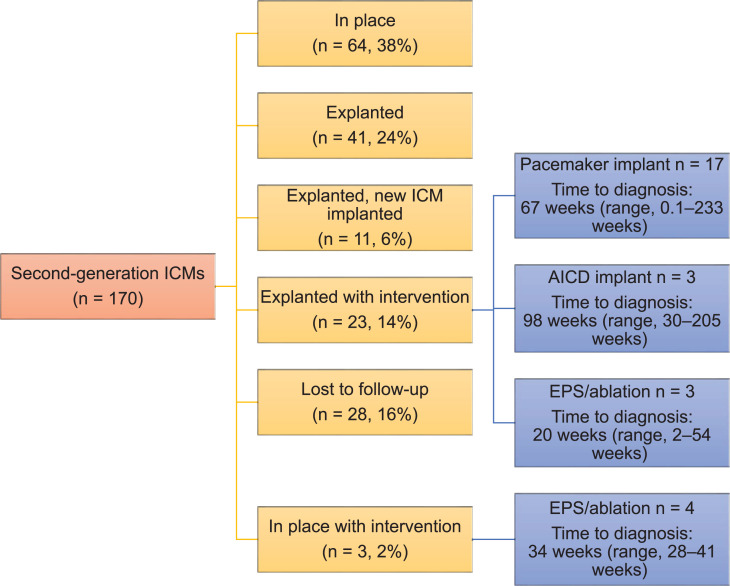
Follow-up of second-generation implantable cardiac monitors (ICMs). One-hundred seventy second-generation ICMs were implanted between 2014–2019. Of these devices, 38% are currently in place, 24% were explanted, 6% were explanted with a new ICM implanted, 14% were explanted with an intervention (pacemaker implant, automatic implantable cardioverter-defibrillator implant, or electrophysiology study/ablation), 16% were lost to follow-up, and 2% were left in place with an intervention (electrophysiology study/ablation). *Abbreviations*: AICD, automatic implantable cardioverter-defibrillator; EPS, electrophysiology study; ICM, insertable cardiac monitor.

**Figure 4:**
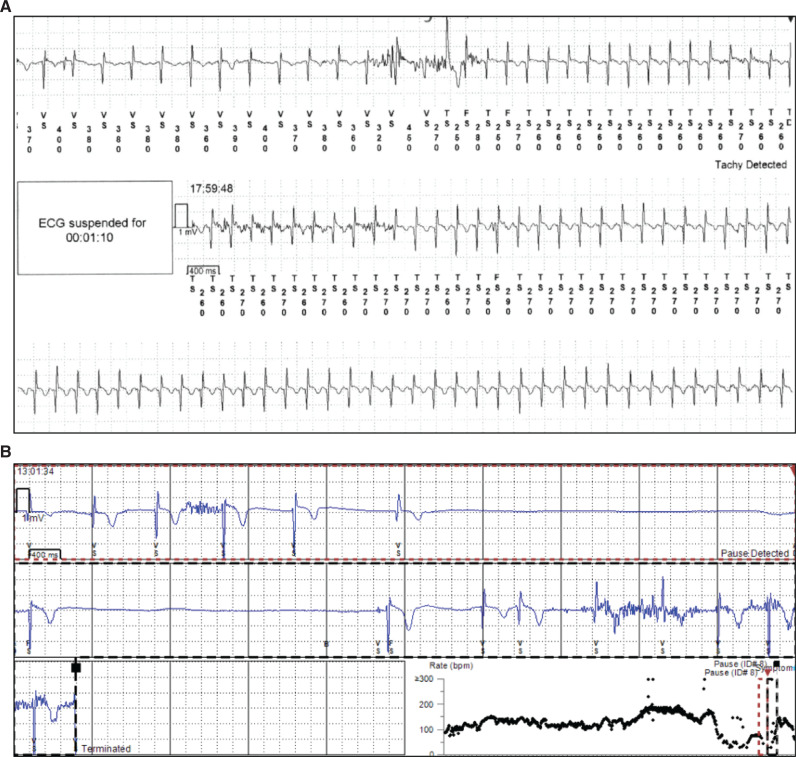

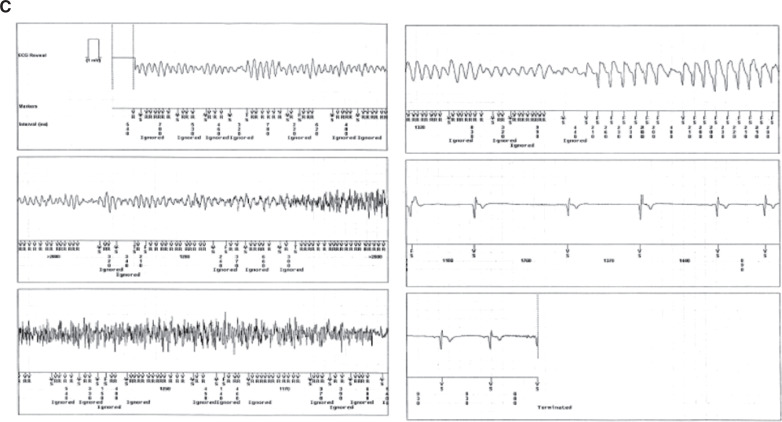
Representative tracings from 3 patients who proceeded to further interventions. **A:** This 2-year-old girl presented with paroxysmal palpitations and this rhythm was documented during long-term implantable cardiac monitor (ICM) monitoring and an episode of palpitations. The patient was taken to the electrophysiology laboratory and had her supraventricular tachycardia ablated. The ICM was removed during the same procedure. Clinically, the patient has done well since then. **B:** This 5-year-old girl underwent ICM implantation for infrequent syncope. During ICM remote monitoring, the patient had an episode of syncope and a sinus pause of 3.2 seconds was documented. The device captured the episode as both a symptom-triggered event and an automatic recorded event. The patient was contacted and underwent transvenous single-chamber implantation with ICM removal the following day. **C:** This 13-year-old boy presented with infrequent exercise-induced syncope and premature ventricular complexes on baseline electrocardiogram and an otherwise normal family history. During this event, the patient had been driving and was stopped by the police for speeding. He had a syncopal event while on the side of the road after he had exited the vehicle. The ICM documented torsades de pointes, which spontaneously terminated. After transmission of these data, the patient was admitted to the hospital for implantation of an automatic implantable cardioverter-defibrillator, which was completed the next day after exercise testing demonstrated an increased premature ventricular complex burden with activity and peak exercise consistent with catecholaminergic polymorphic ventricular tachycardia. Several months later, genetic testing was performed, which provided a genetic confirmation of catecholaminergic polymorphic ventricular tachycardia.

Thirty-seven (18%) patients were lost to follow-up (first-generation, n = 9, 24%; second-generation, n = 28, 16%; *P* = not significant). The majority of first-generation ICMs were explanted (n = 21, 55%). Eight patients (21%) proceeded to have a second intervention, including a pacemaker implant (n = 4, 11%), an ICD implant (n = 1, 3%), an EPS with catheter ablation (n = 1, 3%), and a new ICM implant (n = 2, 5%) **(Figure 2 and Tables 3 and 4)**. The average time to intervention with first-generation ICM was 76 weeks (range, 1.9–191 weeks) **(Figure 2)**. In patients with second-generation ICMs, 64 (38%) had the original ICM in place; 41 (24%) had had an ICM explanted; and 38 (22%) had an additional intervention, including a pacemaker implant (n = 17, 10%), an ICD implant (n = 3, 2%), an EPS + ablation (n = 7, 4%), and a new ICM implant (n = 11, 6%). The average time to intervention was 74 weeks (range, 1–240 weeks) **(Figures 3 and 4 and Tables 3 and 4)**.

### Complications

Complications were seen in both groups, occurring in 5% of first-generation versus 4% of second-generation ICM patients (*P* = .9) **(Table 5)**. Importantly, erosions were only seen early on in the second-generation ICM implant experience (n = 2/20, 10%) and occurred early in the implant experience with these devices (≤6 months). During this time, devices were not sutured into position, and closure was done with topical skin adhesive. This resulted in a change in our clinical practice with the implanting electrophysiologist placing 1–2 subcutaneous sutures to better approximate the incision; following that practice change, no further device erosions were seen in 150 implants.

### Inherited arrhythmia/cardiomyopathy syndrome patients

There was a significant increase in the prevalence of IAS diagnosis among patients implanted with second-generation ICMs compared to those implanted with first-generation ICMs, with IAS patients making up 24% of second-generation ICM recipients versus only 5% of first-generation ICM recipients **(Table 2)**. The majority of IAS patients (n = 28, 78%) were genotype-positive, but 8 (22%) patients were either genotype-negative or -untested/-unknown. Indications for initial implant included long-term arrhythmia surveillance (n = 33, 92%) and liberalization of guideline-recommended activity restrictions (n = 3, 8%). When ICMs reached end of life, 6 patients underwent ICM replacement (8%). Two patients had an upgrade of their ICMs to other cardiac devices, 1 to a pacemaker (genotype-positive LQTS type 3, documented intermittent high-grade atrioventricular block by ICM) and 1 to an automatic ICD (genotype-positive LQTS type 2, documented torsades de pointes on ICM despite optimal medical management).

## Discussion

In this study, we quantified the significant increase in the utilization of second-generation ICMs compared to first-generation ICMs in a pediatric population. Additionally, we demonstrated that the clinical practice and utility for implantation may be broader than current guidelines would indicate,^16,17^ specifically with an increased use of second-generation ICMs in patients with IAS. Broad clinical adaptation within our institution was multifactorial, including a miniaturized form factor and ease of insertion.

The increased utilization of second-generation ICMs in patients with IAS is an important shift in our practice. While first-generation ICMs were predominantly used for pediatric patients with syncope and palpitations,^9^ these data show that second-generation ICMs had an increased usage, with a 5-fold increase in use for IAS patients (5% first-generation vs. 24% second-generation). The main reasons for implanting ICMs in IAS patients were for return to sports clearance (based on the physician’s preference) and arrhythmia surveillance. It is also important to note that a smaller proportion of second-generation ICMs were implanted for syncope or near-syncope (71% first-generation vs. 48% second-generation).

One factor that likely influenced the increased utilization of second-generation ICMs was the miniaturization in form factor, which was particularly important in our pediatric population **(Table 6)**. Second-generation ICMs had a 6-fold reduction in device volume and a 5-fold reduction in device weight. Additionally, second-generation ICMs were half the length and 6.5-fold less broad than the first-generation ICMs. Despite this miniaturization in form factor, these data did not show a significant shift toward use in younger/smaller patients.

Another influencing factor was faster insertion due to the smaller form factor described above, coupled with the custom implant tools, both of which likely contributed to greater utilization. Previous studies have shown that the second-generation ICM implant technique has led to shorter procedure and recovery times as well as increased use within pediatric cardiology.^18^ Anesthetic use may also contribute to increased insertion rates of second-generation ICMs, with Bezzerides et al. reporting that the most frequent type of anesthesia for ICM implants in pediatric patients was conscious sedation (50% of patients), followed by general anesthesia (41%) and local anesthesia (16%).^10^

The average time to diagnosis for both first- and second-generation devices solidifies the need for ICMs in the pediatric population. The average time to diagnosis was 38 weeks for patients with first-generation devices and 55 weeks for those with second-generation devices, demonstrating that some patients require long-term monitoring solutions.^19,20^ The longer duration in the second-generation group may reflect the increased use in IAS patients who required long-term arrhythmia monitoring. In these cases, or in cases with infrequent symptoms, external monitors or wearables may not be the best option. External monitors also pose an issue for young children and toddlers due to skin reactions to the adhesive or intolerance to prolonged monitor wearing. For patients whose symptoms occur during physical activity, there may be difficulty with adherence of patches or perceived interference with performance, causing non-compliance of device use. While external monitors and wearables remain an excellent modality for a short period of time, ICMs may be a more reasonable option for longer-term monitoring.^21^

Lastly, there was no difference noted in complication rates between first-generation and second-generation implants (5% vs. 4%), which would affect practice. After 2 patients with second-generation ICM implants experienced device erosions, the closure technique was modified to include 1–2 subcutaneous sutures to tightly approximate the incision.^18^ Following this change, there were no further erosions documented.

### Study limitations

This is a single-center study and therefore reflects the selection bias of the pediatric electrophysiologists at this center. Given the small sample size of first-generation devices compared to the larger sample size of second-generation devices, it is difficult to make statistically significant conclusions between the groups. In addition, a substantial percentage of patients were lost to follow-up, a consequence of the retrospective study design.

## Conclusion

In our population of pediatric patients, the use of ICMs has evolved dramatically, with both a rapid increase in annual implant volume and increasingly common use in patients with IASs. While we did not assess the attitudes of implanting clinicians, we speculate that the overall increased utilization of ICMs can be at least partly attributed to the smaller form factor and ease of implant. In our practice, second-generation ICMs have become a useful diagnostic and long-term monitoring tool in pediatrics.

## Figures and Tables

**Table 1:** Demographic Data

Demographic Data	First-generation ICMs (n = 38)	Second-generation ICMs (n = 170)	*P* Value
Sex			
Female	17 (45%)	91 (53.5%)	
Male	21 (55%)	79 (46.5%)	
Age, years	13.3 ± 4.7	13.3 ± 4.8	1.0
Weight, kg	57.7 ± 28.6	53.0 ± 24.8	.4
Height, cm	155.3 ± 25	155.3 ± 27	.5
Device model	MDT Reveal Dx 31 (82%) MDT Reveal XT 7 (18%)	MDT LINQ 151 (89%) SJM Confirm 19 (11%)	

*Abbreviations:* ICM, insertable cardiac monitor; MDT, Medtronic (Minneapolis, MN, USA); SJM, St. Jude Medical (St. Paul, MN, USA).

**Table 2:** Indications for Implant

Indication(s) for Implant	First-generation ICMs (n = 38)	Second-generation ICMs (n = 170)	*P* Value
Syncope/near syncope	27 (71%)	81 (48%)	.009
Palpitations	6 (16%)	32 (19%)	.66
Inherited arrhythmia syndrome	2 (5%)	40 (24%)	.01
PVC/VT	3 (8%)	11 (6%)	.75
Atrial fibrillation	0	2 (1%)	.5
Tachycardia	0	3 (2%)	.4
Heart block	0	1 (0.5%)	.64

Indications for implant are compared between first- and second-generation ICMs. There was a higher percentage of first-generation ICMs implanted for syncope and near-syncope (*P* = .009) versus a higher percentage of second-generation ICMs implanted in patients with inherited arrhythmia syndromes (*P* = .01).

*Abbreviations*: ICM, insertable cardiac monitor; PVC, premature ventricular complexes; VT, ventricular tachycardia.

**Table 3:** Number of Interventions*

Total Number of Interventions	First-generation ICMs (n = 38)	Second-generation ICMs (n = 170)
**Diagnosis leading to pacemaker implant**	**4**	**17**
Syncope + sinus pause (structurally normal heart)	0	14
High-grade AV block + LQTS 3	0	1
Sinus pause + s/p ASO for D-TGA	0	1
Sinus pause + s/p AVC repair	0	1
**Diagnosis leading to AICD implant**	**1**	**3**
Ventricular tachycardia	0	2
Torsades de pointes	1	1
**Diagnosis leading to EP study ± ablation**	**1**	**7**
SVT in a structurally normal heart	1	5
SVT s/p TOF repair	0	1
Atrial fibrillation	0	1
**Diagnosis leading to a new ICM implant**	**3**	**11**
Syncope	2	2
Palpitations	0	2
Ventricular tachycardia	0	1
Inherited arrhythmia syndrome management	1	6
**Total**	**9 (24%)**	**38 (22%)**

*The number of patients who underwent a pacemaker placement, AICD implantation, or EP study with or without ablation.

*Abbreviations*: AICD, automatic implantable cardioverter-defibrillator; ASO, arterial switch operation; AVC, atrioventricular canal; D-TGA, d-looped transposition of the great arteries; EP, electrophysiology; ICM, insertable cardiac monitor; LQTS, long QT syndrome; SVT, supraventricular tachycardia.

**Table 4:** Summary Data for Secondary Interventions on Patients Implanted with ICM for Syncope/Near-syncope and Palpitations

Patients Implanted for Syncope/Near-syncope or Palpitations	First-generation ICMs (n = 31)	Second-generation ICMs (n = 111)
PM implantation following ICM	4	14
AICD implantation following ICM	1	2
EPS following ICM	1	4
New ICM monitor placed at initial ICM EOL	2	4
Diagnostic yield	26% (8/31)	20% (22/111)

*Abbreviations*: AICD, automatic implantable cardioverter-defibrillator; AVC, atrioventricular canal; CPVT, catecholaminergic polymorphic ventricular tachycardia; EOL, end of life; EPS, electrophysiology study; ICM, insertable cardiac monitor; LQTS, long QT syndrome; PM, pacemaker; TGA, transposition of the great arteries; TOF, tetralogy of Fallot; s/p, status post; VT, ventricular tachycardia; WPW, Wolff–Parkinson–White. There were 31 first-generation ICM patients implanted for syncope/near-syncope and palpitations. From this cohort, there were 8 patients who required a second intervention: 4 patients required a PM implantation for documented, symptomatic sinus pauses/asystole, 1 patient required an AICD implantation for documented torsades de pointes (and was subsequently diagnosed with catecholaminergic polymorphic tachycardia), 1 patient had an EPS (this patient had an EPS + ablation for WPW and subsequently had an ICM placed, then underwent a second EPS, which was negative), and 2 patients had a repeat ICM implant (1 patient with intermittent exertional syncope and the other with continuing palpitations and syncope s/p EPS ×2). From this, our yield from the first-generation ICMs in syncope/near-syncope and palpitation patients numbered 8/31, or 26%. From the second-generation ICM patients, there were 24 patients who required a second intervention: 14 patients required PM implantation (12 with symptomatic sinus pauses, 1 patient s/p AVC repair with documented sinus pause and syncope on ICM, and 1 patient s/p arterial switch for D-TGA and documented pause on ICM), 2 patients required ICD implantation (both patients had documented symptomatic VT on the ICM, but 1 patient was later diagnosed with LQTS and the other with CPVT), 4 patients went on to undergo an EPS (2 patients had documented SVT, 1 patient s/p TOF repair had documented SVT, and 1 patient had documented atrial fibrillation), and 4 patients had a new ICM placed at the time of previous ICM explant (1 for asymptomatic sinus pauses, 1 for pocket infection, 1 for discomfort at implant site, and 1 for recurrent palpitations). The yield for the second-generation ICM group was 22%.

**Table 5:** Complications

Complications	First-generation ICMs (n = 38)	Second-generation ICMs (n = 170)	*P* Value
Infection	0	2 (1%)	.5
Pain at implant site	2 (5%)	4 (2%)	.3
Erosion	0	2 (1%)	.5
Total	2 (5%)	8 (4%)	.9

*Abbreviation*: ICM, insertable cardiac monitor. While there was no statistically significant difference in complication rates, there were different types of complications noted with the second-generation ICMs likely attributable to the implant technique.

**Table 6:** Device Sizes

	Reveal XT	Reveal LINQ	Confirm
Volume, mL	9	1.2	1.4
Dimensions, mm	95 × 62 × 8	44.8 × 7.2 × 8	49 × 9.4 × 3.1
Weight, g	15	2.5	3

This table shows a comparison of the volume, dimensions, and weight for each ICM, including the Medtronic Reveal XT ICM (first-generation), Medtronic Reveal LINQ (second-generation), and Abbott Confirm (second-generation). Comparing the first-generation ICM and second-generation ICM demonstrates the significantly smaller form factor of the second-generation devices.
